# On the Role of Peripheral Sensory and Gut Mu Opioid Receptors: Peripheral Analgesia and Tolerance

**DOI:** 10.3390/molecules25112473

**Published:** 2020-05-26

**Authors:** Susanna Fürst, Zoltán S. Zádori, Ferenc Zádor, Kornél Király, Mihály Balogh, Szilvia B. László, Barbara Hutka, Amir Mohammadzadeh, Chiara Calabrese, Anna Rita Galambos, Pál Riba, Patrizia Romualdi, Sándor Benyhe, Júlia Timár, Helmut Schmidhammer, Mariana Spetea, Mahmoud Al-Khrasani

**Affiliations:** 1 Department of Pharmacology and Pharmacotherapy, Faculty of Medicine, Semmelweis University, Nagyvárad tér 4, P.O. Box 370, H-1445 Budapest, Hungary; zadori.zoltan@med.semmelweis-univ.hu (Z.S.Z.); zador.ferenc@pharma.semmelweis-univ.hu (F.Z.); kiraly.kornel@med.semmelweis-univ.hu (K.K.); mbalogh@mdanderson.org (M.B.); laszlo.szilvia@med.semmelweis-univ.hu (S.B.L.); hutka.barbara@semmelweis-univ.hu (B.H.); mohammadzadeh.amir@med.semmelweis-univ.hu (A.M.); galambos.anna@pharma.semmelweis-univ.hu (A.R.G.); riba.pal@med.semmelweis-univ.hu (P.R.); timar.julia@med.semmelweis-univ.hu (J.T.); al-khrasani.mahmoud@med.semmelweis-univ.hu (M.A.-K.); 2 Institute of Biochemistry, Biological Research Center, Temesvári krt. 62., H-6726 Szeged, Hungary; benyhe.sandor@brc.hu; 3 Department of Pharmacy and Biotechnology (FaBiT), Alma Mater Studiorum University of Bologna Via Irnerio 48, 40126 Bologna, Italy; chiara.calabrese2@studio.unibo.it (C.C.); patrizia.romualdi@unibo.it (P.R.); 4 Department of Pharmaceutical Chemistry, Institut of Pharmacy and Center for Molecular Biosciences Innsbruck (CMBI), University of Innsbruck, Innrain 80-82, 6020 Innsbruck, Austria; helmut.schmidhammer@uibk.ac.at (H.S.); mariana.spetea@uibk.ac.at (M.S.)

**Keywords:** peripheral µ-opioid receptors, analgesia, peripheral analgesic tolerance, dysbiosis

## Abstract

There is growing evidence on the role of peripheral µ-opioid receptors (MORs) in analgesia and analgesic tolerance. Opioid analgesics are the mainstay in the management of moderate to severe pain, and their efficacy in the alleviation of pain is well recognized. Unfortunately, chronic treatment with opioid analgesics induces central analgesic tolerance, thus limiting their clinical usefulness. Numerous molecular mechanisms, including receptor desensitization, G-protein decoupling, β-arrestin recruitment, and alterations in the expression of peripheral MORs and microbiota have been postulated to contribute to the development of opioid analgesic tolerance. However, these studies are largely focused on central opioid analgesia and tolerance. Accumulated literature supports that peripheral MORs mediate analgesia, but controversial results on the development of peripheral opioid receptors-mediated analgesic tolerance are reported. In this review, we offer evidence on the consequence of the activation of peripheral MORs in analgesia and analgesic tolerance, as well as approaches that enhance analgesic efficacy and decrease the development of tolerance to opioids at the peripheral sites. We have also addressed the advantages and drawbacks of the activation of peripheral MORs on the sensory neurons and gut (leading to dysbiosis) on the development of central and peripheral analgesic tolerance.

## 1. Introduction

The present consensus is that all opioid agonists used in clinical practice produce analgesia primarily mediated by µ-opioid receptors (MORs) located within the brain and spinal cord along the pain transmission pathways. On the other hand, the adverse effects of opioids are also mediated through the activation of opioid receptors (ORs), both in the central nervous system (CNS) and in the periphery [1,2,3]. There are four opioid receptor types named as MORs, δ-opioid receptors (DORs), κ-opioid receptors (KORs), and the nociceptin opioid receptor that have been cloned and extensively pharmacologically characterized [4,5,6,7,8,9,10,11,12,13]. ORs belong to the large family of G protein-coupled receptors (GPCRs) [14,15]. The binding of opioid agonists to central and peripheral ORs initiate signaling downstream events that lead to the activation of G_i/o_ proteins, β-arrestin recruitment, opening of G protein-coupled inwardly rectifying potassium (GIRK) channels and the inhibition of voltage-gated Ca^2+^ channels [10,14,16,17]. Concurrently, besides receptor-type selectivity, the central versus peripheral distribution of opioid receptors and their functional relevance have gained increased attention in the opioid research. ORs are distributed on key points involved in the modulation of nociception along the ascending and descending pain pathways [3,18]. Unfortunately, the activation of ORs in the CNS and in the periphery results in the occurrence of undesirable side effects. Centrally mediated adverse effects of opioid analgesics include respiratory depression, sedation, nausea, and dizziness, while constipation predominantly results from the activation of intestinal opioid receptors. The development of analgesic tolerance, together with abuse potential, often limit the clinical utility of MOR analgesics leading to early discontinuation, under-dosing, and inadequate analgesia in pain patients.

At present, in the clinical setting, the only option available to overcome the development of analgesic tolerance is to increase the dose (escalation to higher dose) or to use other opioid analgesics to maintain analgesia (opioid rotation). The consequence of increasing opioid dose is exposing the patient to the risk of adverse events, including overdose as well as the misuse of and addiction potential to opioids [19]. It is well-known that opioid analgesic tolerance develops to all clinically used opioids; however, the degree and the rate of tolerance to the opioid side effects depends on the target that hosts the opioid receptors. For example, opioid tolerance develops more rapidly to the analgesic effects, whereas little tolerance manifests to constipation [20]. This phenomenon is described as differential tolerance development [21]. In a clinical setting, it means that analgesic tolerance is rapidly developed compared to the development of tolerance to gastrointestinal (GI) side effects (constipation, if it occurs at all). In addition, along the GI tract, there are regional differences in opioid tolerance development, namely in the upper GI tract tolerance can develop to the motility, whereas in the colon such is not the case, which is reflected by a persistent constipation following chronic treatment with opioids [22]. From these observations, it could be concluded that opioid tolerance development is the fastest and most profound for the analgesic actions, less for the respiratory depressant effects and least for GI motility [21,22,23].

Physicians’ view on opioid euphoric tolerance is that drug abusers are seeking a higher opioid dose in order to maintain the euphoric effect. This dose escalation might lead to overdose deaths, which is the major cause contributing to the current opioid epidemic [24,25,26,27]. In humans addicted to morphine, tolerance reaches an extreme degree, with doses of 300–600 mg (30–60 times the normal dose) often being taken several times a day [20,28,29].

The magnitude of analgesic tolerance also depends on the pharmacological profiles of opioids; an important one is the agonist efficacy, but also the pharmacokinetic profiles such as the route of administration. Though in the latter case, it has been reported that in tolerant subjects, the pharmacokinetic parameters of morphine such as absorption, metabolism, and excretion were unaffected [30]. Nevertheless, the dosing, the intervals, and routes of administration, the applied methodologies, as well as the animal species have been reported to substantially affect the development of analgesic tolerance [28,29,31].

The putative target for opioid analgesics currently available in clinical practice is the CNS, which hosts many targets that mediate analgesia and other effects including analgesic tolerance, respiratory depression, and addiction liability. The growing evidence on the existence of functional peripheral ORs, particularly MORs has initiated research efforts to develop opioid analgesics with limited CNS penetration in order to gain analgesia free of the central unwanted side effects [2,26,32,33,34,35,36,37,38,39]. On the other hand, recent studies have also suggested that MORs that are distributed in the periphery on dorsal root ganglia (DRG) or GI tract are implicated in the development of analgesic tolerance [40,41].

In this review, we focus on the MORs-mediated peripheral analgesia in different animal pain models. Next, the contribution of MORs and drawbacks upon their activation to opioid-induced analgesic tolerance is discussed. The review also briefly discusses the following questions: (i) Besides peripheral analgesia, does the activation of MORs located on the sensory afferent neurons influence the central analgesic effect of opioids? (ii) What is the role of MORs’ activation in the alteration of gut microbiota and whether or not these changes can contribute to the development of central analgesic tolerance? (iii) What is the current view on the role of microbiota alteration in the development of peripheral analgesic tolerance?

## 2. Contribution of Peripheral MORs to Opioid-Induced Analgesia and Analgesic Tolerance

There is substantial evidence demonstrating the involvement of peripheral ORs in MOR agonists-induced analgesia following systemic administration in acute thermal pain models in rat or mouse [34,42]. Research from our laboratory (Schmidhammer and Spetea’s, Al-Khrasani and Fürst’s and Benyhe’s research teams) and others have reported on effective, peripheral analgesic effects of opioids following local or systemic administration applying various pharmacological approaches in rodent models of inflammatory and visceral pain [2,35,36,37,38,39,43,44,45,46,47,48,49,50,51,52,53]. On the other hand, in animal models of neuropathic pain, the peripheral analgesic effect of opioids is a question of debate [54,55,56,57,58,59]. Animal studies that examine peripheral analgesic effects following systemic administration of peripherally restricted MOR agonists and consideration of time-dependent changes of MORs reserve and analgesia peri-induction of neuropathic pain will reopen old avenues in this field of pain research.

In opioid research as well as other drug discovery areas, the limited access of substances to the CNS can be achieved by either quaternization of the molecule or the introduction of functional groups endowing the molecule to have a zwitterionic structure at body pH (Table 1) [34,35,36,37,47,60,61,62,63,64,65]. The number of MOR agonists with limited CNS penetration and displaying peripheral analgesia have been increasing over the years, but they have not been proven so far to be of clinical value [43,52,66,67]. In addition, these results may initiate further studies to examine the development of peripheral analgesic tolerance, since most research has been focused on the evaluation of the central opioid analgesic tolerance. For examination of peripheral analgesia, studies have shed light on the peripheral analgesic tolerance [68,69]. Research on the development of the centrally mediated opioid analgesic tolerance and related mechanisms has been reviewed extensively elsewhere [20,23,70,71,72]. Available evidence related to peripheral analgesia and analgesic tolerance is summarized below. This section reviews the evidence related to peripheral analgesia and analgesic tolerance according to the animal pain models studied, namely (1) acute thermal, (2) acute and subchronic inflammatory, and (3) neuropathic.

### 2.1. Peripheral Opioid Analgesia and Tolerance in Animal Models of Acute Thermal Pain

The group of G.W. Pasternak has reported on the analgesic effects of morphine, [D-Ala^2^, N-Me-Phe^4^,-Gly-ol^5^]enkephalin (DAMGO), and morphine-6β-glucuronide (M6G) following local administration to the mouse tail in the tail-flick assay, which is an acute thermal pain model [74]. In this study, the intrathecal administration of antisense targeting exons 1 and 4 of MOR-1 blocked the local analgesic effect of morphine, indicating the involvement of the terminals of sensory neurons. Moreover, the repeated systemic administration of morphine or repeated daily exposure of the tail to morphine caused profound analgesic tolerance to the local analgesic effect of morphine [75]. Systemic or local but not intrathecal MK801, an N-methyl-D-aspartate (NMDA) receptor antagonist, abrogated morphine-induced peripheral analgesic tolerance when morphine was applied superficially in DMSO solution. One of the key observations of this study regarding the peripheral analgesic tolerance is that peripheral NMDA receptors are implicated in the development of analgesic tolerance to topically applied morphine. Notably, the same group also showed mixed results in terms of cross-tolerance, which occurred only between morphine and DAMGO, but not between morphine and M6G. Furthermore, M6G but not morphine or DAMGO produced 3-methoxynaltrexone sensitive local analgesic effect, indicating a unique mechanism of action of M6G. It has also been reported that beside MK-801, ketamine was able to suppress tolerance development. These data are in agreement with other studies where NMDA receptor antagonists inhibited the development of opioid analgesic tolerance, despite the fact that the sites of actions are different. In addition, the involvement of NMDA receptors in neural plasticity explains the effectiveness of NMDA receptor antagonists in the inhibition of central opioid analgesic tolerance, as described by many research groups [74,75,76,77,78,79]. Nevertheless, the existence of NMDA receptors on peripheral sensory neurons is well-documented [80,81,82], but the role of these receptors on peripheral opioid analgesic tolerance remains unclear.

DRG is the MORs’ synthesis machinery of primary sensory neurons, and any changes in DRG-related MORs are able to alter the magnitude of analgesia evoked by peripherally administered opioid agonists. Meuser and coworkers [83] showed that systemic morphine treatment (4 days 2 × 10 mg/kg) caused naloxone reversible downregulation of MOR mRNA in the DRG of rats that developed morphine analgesic tolerance in the hot-plate test. The repeated treatments resulted in the decrease in the expression of MORs in the DRG, which might contribute to a reduction in peripheral analgesia. Aδ and C primary sensory afferent fibers convey the pain from the site of injury into the spinal cord, and the decrease in functional MORs can affect the pain intensity. In this work, the authors used an acute thermal pain model, where the MORs’ reserve upon the induction of pain remains unchanged [83]. Sun and co-workers developed conditional knock-out (KO) mice, in which the expression of the MOR gene (Oprm1) was entirely abolished in DRGs and substantially decreased in the spinal cord [84]. In the Oprm1 conditional KO animals, systemic or intrathecal morphine treatment showed limited effect in acute thermal and mechanical pain. In addition, opioid-induced hyperalgesia (OIH) following chronic morphine treatment was completely absent in these animals. In addition, systemic morphine treatment showed weak analgesic effect in these conditional KO animals that hampered assessing analgesic tolerance as well.

**Figure 1:**
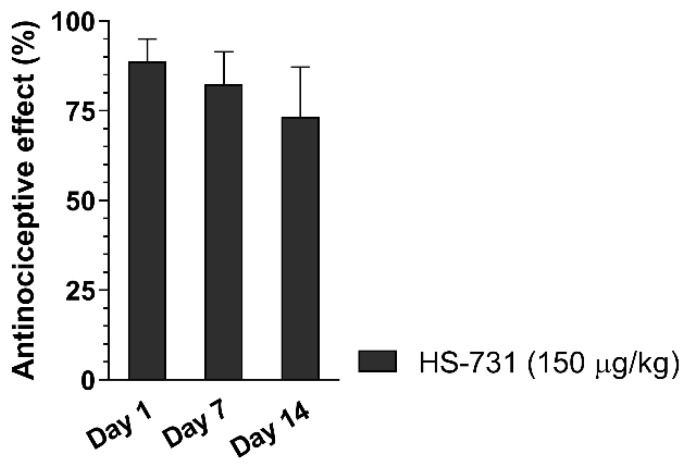
Effect of chronic treatment on the development of peripheral analgesic tolerance of HS-731 in the rat tail-flick test after systemic (s.c.) administration. Rats were s.c. administered daily for two weeks HS-731 (150 µg/kg). Antinociceptive effects were measured at days 1, 7, and 14, at 60 min after administration of HS-731. There was no significant effect day 7 vs. day 1 and day 14 vs. day 1. Statistical differences were determined with one-way ANOVA and Newman–Keuls multiple comparisons post-hoc test. Data represent means ± S.E.M (n = 7 per group). Experiments were performed and analyzed as described previously [62]. Tolerance protocol was developed by Fürst and Spetea.

Work carried out by the groups of Fürst, Spetea, and Schmidhammer has reported on the involvement of peripheral ORs in mediating the antinociceptive effect of systemically (subcutaneous, s.c.) administered 6β-glycine substituted 14-*O*-methyloxymorphone, HS-731 (Table 1), which is an MOR selective agonist with limited CNS penetration in a rat model of acute thermal (tail-flick test) pain [34]. The antinociceptive efficacy of HS-731 resulting from the activation of peripheral ORs was also demonstrated in rat models of inflammatory pain (formalin test and carrageenan-induced hyperalgesia) [34,35], in a mouse model of visceral pain (acetic acid-induced writhing assay) [37,51], and in the mouse eye-wiping trigeminal nociceptive test [36]. Other 6-amino acid- and 6-dipeptide-substituted derivatives of 14-*O*-methyloxymorphone derivatives were also reported to produce peripherally-mediated antinociception after systemic (s.c.) administration in rats and mice [37]. The effect of repeated systemic (s.c.) treatment of rats with HS-731 on the development of analgesic tolerance was measured in the tail-flick test. Daily treatment of rats for 14 days resulted in no analgesic tolerance for HS-731 (Figure 1) (Fürst and Spetea, unpublished data). This finding provides clear evidence that the selective activation of peripheral ORs leads to effective antinociceptive effects without resulting in analgesic tolerance following systemic opioid administration.

**Figure 2:**
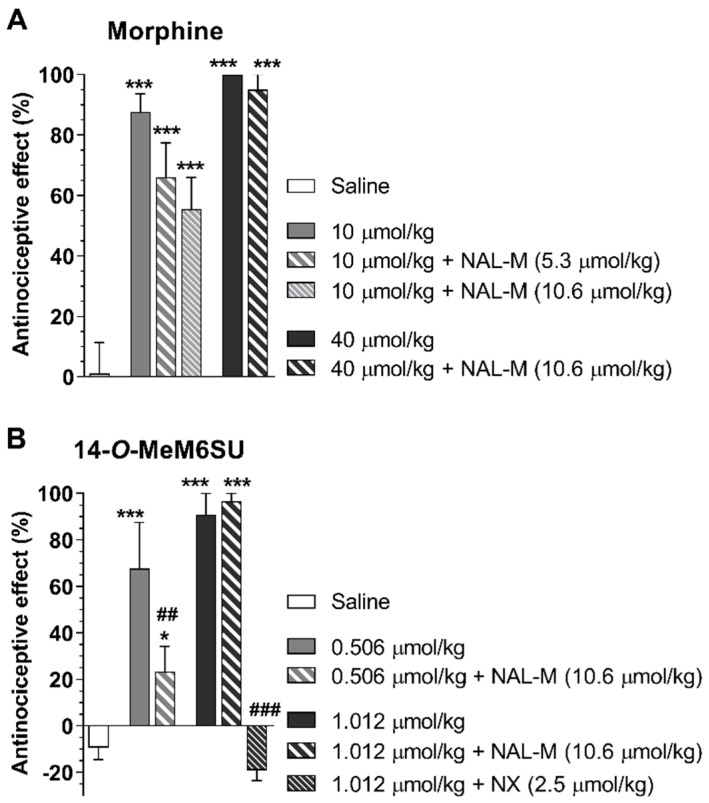
The analgesic effect of morphine (**A**) and 14-*O*-methylmorphine-6-*O*-sulfate (14-*O*-MeM6SU; **B**) in the rat tail-flick test at 30 and 60 min, respectively after s.c. administration. Their effects were assessed in the presence of naloxone methiodide (NAL-M), which is a peripherally acting OR antagonist. The analgesic effect of the highest dose 14-*O*-MeM6SU was abolished by the OR antagonist naloxone (NX). (*p < 0.05 and ***p < 0.001, compared to saline group; ##*p* < 0.05 and ###*p* < 0.001, compared to 14-O-MeM6SU alone). Statistical differences were determined with one-way ANOVA and Newman–Keuls multiple comparisons post-hoc test. Data represent means ± S.E.M (n = 3–9 per group). Experiments were performed and analyzed as described previously [62].

In another study, 14-*O*-methylmorphine-6-*O*-sulfate (Table 1), a novel selective MOR agonist [62], proved to produce peripheral antinociceptive effects in rats in the tail-flick test following s.c. administration (Al-Khrasani et al., unpublished data, Figure 2). However, the peripheral effect of 14-*O*-methylmorphine-6-*O*-sulfate was measured only after administration of low doses, since at higher doses, a central analgesic effect was observed. In addition, it was proven that this molecule produced less analgesic tolerance than morphine in mice, although the applied doses in these experiments were high enough to produce a central effect [85]. No study on the peripheral analgesic tolerance of 14-*O*-methylmorphine-6-*O*-sulfate has been reported so far.

### 2.2. Peripheral Opioid Analgesia and Tolerance in Animal Acute and Subchronic Inflammatory Pain Models

Aley and coworkers [86] found that intradermal co-injection of the highly selective MOR agonist peptide DAMGO and the adenosine A1-receptor agonist, N6-cyclopenthyladenosin, into the rat hind paw can dose-dependently inhibit PGE2 induced hyperalgesia. Repeated administration of both compounds (3x hourly) caused a rapid and cross-tolerance development. In addition, either naloxone or the A1 receptor antagonist PACPX caused (cross) withdrawal hyperalgesia in pretreated animals. Co-treatment with the receptor antagonists mentioned above blocked the development of tolerance and agonist-induced hyperalgesia. The same group [76] reported that NO generation and protein kinase C (PKC) activation are contributing to the development of analgesic tolerance and withdrawal hyperalgesia, respectively.

Honoré and coworkers [87] investigated spinal c-Fos expression and pressure hyperalgesia following systemic (i.v.) or intraplantar (i.pl.) morphine treatment in a carrageenan-induced pain model. Morphine given systemically (i.v.) or locally (i.pl.) blocked carrageenan-induced increase in spinal c-Fos expression and the development of hyperalgesia, which was indicated by a decrease in paw pressure threshold. These behavioral effects were abolished when animals were subjected to three days pretreatment with s.c. morphine (80 mg/kg).

Among the most established chemical nociceptive stimuli, bradykinin has been reported to be an important mediator of pain [88]. The MOR agonist morphine showed a naloxone-reversible analgesic effect on bradykinin-induced pain in mice in a study carried out by Tokuyama and co-workers [89]. In addition, the effect of morphine was localized in the affected limb, since systemic or local administration to the contralateral limb failed to produce analgesia. In this work, the authors have proved that DAMGO and U-69,593, but not [D-Ser^2^,-Leu^5^,-Thr^6^]-enkephalin (DSLET), which are selective agonists for MOR, KOR, and DOR, respectively, were effective in inducing peripheral analgesia [89]. Systemic pretreatment with morphine (5 days, 10 mg/kg) caused tolerance to analgesic effect of s.c. morphine measured by the tail-pinch test, but the effect of local morphine on the bradykinin-induced nociceptive flexion was unaffected. These findings were later confirmed by the same group [90]. On the other hand, they also reported that the repeated local treatment with morphine caused tolerance to its local effect [91]. This tolerance development was effectively blocked by PKC α and γ inhibitors, but less by PKC δ inhibitors.

In inflammatory conditions such as arthritis, peripheral ORs are upregulated [33,92,93,94]. This upregulation was demonstrated to contribute to peripheral opioid analgesia in animals as well as in humans [94,95,96]. The presence of inflammation leads to the release of endogenous mediators that can enhance levels of peripheral ORs’ proteins and mRNA expression and also increased axonal transport and G protein coupling [92,93,97]. Additional changes related to peripheral ORs include increased ORs trafficking and opioid-related actions, such as cyclic adenosine monophosphate (cAMP) accumulation, the modulation of voltage-dependent cation channels etc., and the accessibility to the ORs in the transperineurium of ORs is also enhanced [93,97]. Several studies have also demonstrated that opioid agonists produced pronounced peripheral analgesia in animal models of inflammatory pain [33,98]. Thus, the question is whether the inflammation evoked MORs expression has the ability to affect the development of peripheral analgesic tolerance following repeated peripheral opioid treatment.

Fernandez-Duenas and coworkers [99] investigated the analgesic effect of s.c. morphine in the plantar (Hargreaves test), Randall-Selitto and von Frey tests with or without complete Freund′s adjuvant (CFA)-induced paw inflammation in naive and morphine pellet-exposed mice. They found that acute s.c. morphine treatment resulted in decreased antinociception in mice with CFA-induced paw inflammation compared to mice that were not subjected to CFA treatment. In the plantar test, the antinociceptive effect of morphine was mostly centrally mediated in control animals, but in the case of mice with inflammation, the peripheral analgesic component was increased. On the other hand, the antiallodynic effect of morphine was mainly centrally mediated independent of the presence or absence of inflammation. The potency of morphine was enhanced in mechanical and thermal inflammatory states. Pretreatment with a morphine pellet (3 days from 4 to 7 day after CFA) caused a rightward shift of dose–response curves and a decrease in maximum effect in all test methods [99]. Following chronic morphine treatment, the antinociceptive effect of morphine was the same in mice with or without inflammation, so the relative tolerance was higher in inflammatory conditions. In the paw pressure test, the same research group [100] has also reported the effects of different i.pl.-injected opioids (morphine, fentanyl, buprenorphine, [D-Pen^2^,-D-Pen^5^]enkephalin (DPDPE) and U50488H) and corticotrophin releasing factor (CRF), which is known to induce endogenous opioid release. They demonstrated that all compounds were ineffective in the absence of inflammation, yet in inflamed paws, they were able to increase the pain threshold, achieving a maximal effect of 88% for fentanyl and 48–64% for the rest of the compounds. In morphine-tolerant mice, locally administered morphine failed to produce analgesia, yet a significant decrease in analgesic effects of DPDPE, U50488H, and CRF were indicated by the rightward shift of dose–response curves. In addition, they showed a small decrease in the antinociceptive and maximal effects for fentanyl and no significant change in case of buprenorphine. They concluded that inflammation is required for the local action of opioids and chronic systemic exposure causes (cross) tolerance toward this effect. They investigated further β-arrestin1 and β-arrestin2 expressions in the absence and in the presence of inflammation upon morphine treatment. They found that CFA increases the level of both β-arrestins, which can decrease after acute and chronic morphine exposure, but morphine alone without inflammation caused no change in the expression levels of β-arrestins.

**Figure 3:**
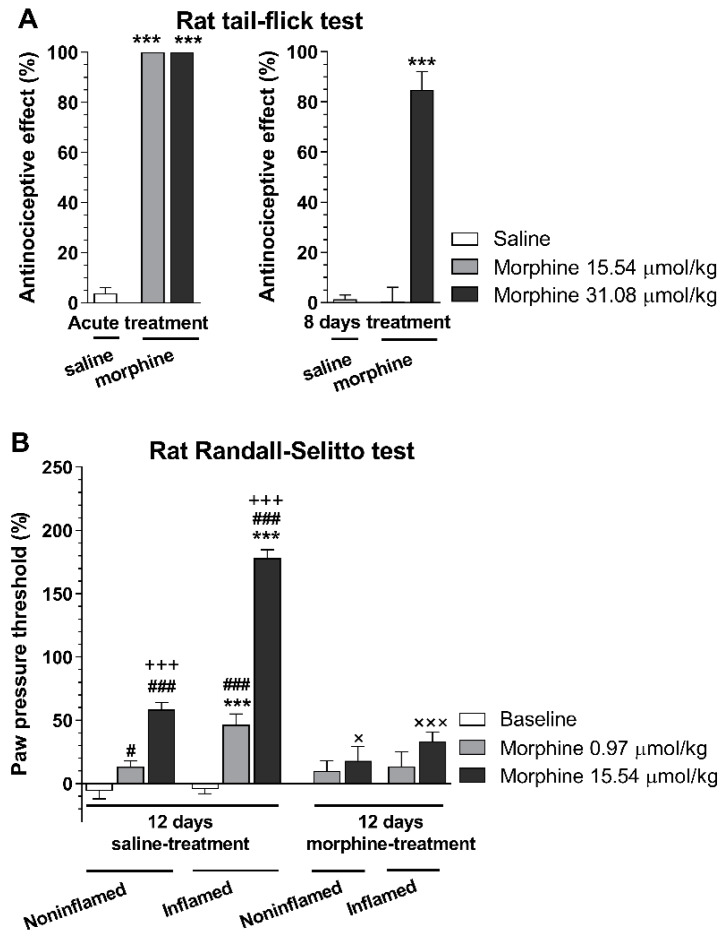
Analgesic effect of morphine in tolerant and non-tolerant rats in the rat tail-flick (**A**) and Randall–Selitto test (**B**). **A**: The analgesic effect in acute treatments was determined 30 min after morphine s.c. injection in the indicated doses. In chronic treatments, animals received 31.08 µmol/kg s.c. twice a day for 8 days. On the 8th day, the antinociceptive effect was determined 30 min after s.c. injection of morphine in the indicated doses (***p < 0.001, saline- vs. morphine-treated group). Statistical difference was determined with one-way ANOVA and Holm–Sidak’s multiple comparison post-hoc test. **B**: Inflammatory pain was induced by complete Freund′s adjuvant (CFA) injection in the hind paw. Animals were subjected to either saline or 31.08 µmol/kg s.c. twice a day for 12 days. On the 8th day, CFA was injected intraplantar (i.pl.) and on 12th day, paw pressure thresholds were determined prior to and 30 min after s.c. injection of morphine in the indicated doses. (***p < 0.001, non-inflamed vs. inflamed paw; #*p* < 0.05 and ###*p* < 0.001, saline vs. morphine treated group within non-inflamed or inflamed paw; +++*p* < 0.001, 0.97 µmol/kg vs. 15.54 µmol/kg morphine group; ×*p* < 0.05 and ×××*p* < 0.001, 12 days saline vs. 12 days morphine within the corresponding groups). Statistical difference was determined with two-way ANOVA, with Holm–Sidak’s multiple comparison post-hoc test. All data represent means ± S.E.M (n = 5–20 per group). Rat tail-flick and Randall–Selitto tests were performed and analyzed as previously described [65], tolerance protocol was developed based on Király et al. [85].

Other investigators [68] showed that the local analgesic effect of i.pl. fentanyl was not affected by 4 days pretreatment with s.c. morphine 10 mg/kg twice daily in CFA-induced inflammatory pain. Such studies raised the question of whether rats subjected to repeated morphine treatment and developed analgesic tolerance in an acute thermal pain model would also develop morphine analgesic tolerance in a subchronic inflammatory pain model. Our (Al-Khrasani’s) research group performed behavioral studies assessing the development of analgesic tolerance in rats with CFA-induced hyperalgesia. The analgesic effect of s.c. morphine was determined using the thermal tail-flick assay and the Randall–Selitto paw pressure test. In these experiments, analgesic tolerance was developed in both non-inflamed and inflamed paws. In addition, there was no difference in non-inflamed and inflamed paw in the effect of morphine in tolerant rats in contrast to non-tolerant animals at higher dose (Figure 3) (Al-Khrasani et al., unpublished data).

In another study carried out by Eidson and Murphy, morphine tolerance was investigated in a rat model of peripheral inflammation (CFA-induced) [101]. They reported that morphine tolerance is accompanied by increased glial cell activation within the ventrolateral periaqueductal gray (vlPAG). Interestingly, persistent peripheral inflammation inhibited the development of morphine tolerance, presumably via the inhibition vlPAG glial activation. This indicates that complex peripheral mechanisms influence the development of analgesic tolerance [33,65].

In a recent study, the combination of loperamide (a peripheral restricted MOR preferring opioid agonist) with the DOR agonist oxymorphindole caused peripheral synergistic analgesia in CFA-induced hyperalgesia in mice [102]. This approach sought to utilize the upregulation of MORs and DORs and their heterodimer formation during inflammation in sensory axons. According to these results, the loperamide–oxymorphindole combination was 150 times more potent systemically and 84 times more effective locally compared to single drug administration. They also concluded that the repeated topical administration of loperamide–oxymorphindole (twice daily for 3 days) did not induce analgesic tolerance in animals with inflammatory pain [102]. Data were assessed by the Hargreaves plantar assay and inflammation was induced by CFA. The majority of the discussed studies suggest the decrease in the peripheral analgesic effects of opioids, although the time lag of chronic treatments, the route of administration, and the efficacy of test compounds are different among these works.

### 2.3. Peripheral Opioid Analgesia and Tolerance in Animal Neuropathic Pain Models

Neuropathic pain management is a clinical challenge of great interest, because not all patients with neuropathic pain respond to the current available medications [103,104,105]. The background mechanisms related to the poor effectiveness of opioids in the treatment of neuropathic pain is still not clarified. One possible explanation is the loss of MORs following nerve injury, which implicates the appliance of higher dosage of opioids, therefore resulting in severe central side effects [56,106,107,108,109]. The functional role of peripheral MORs in peripheral opioid analgesia and analgesic tolerance following the peripheral administration of opioids has not been elucidated yet in neuropathic pain conditions. Early studies used rat peripheral mononeuropathic pain models to assess the analgesic effect of morphine following systemic administration (i.v.). Kayser and coworkers [110] found that low systemic morphine doses produced antiallodynic effect with a mechanism involving the participation of peripheral ORs. In another study, the same group showed that treatment with a higher dose of morphine 10 mg/kg s.c. (twice daily for 4 days) induced complete tolerance to the analgesic effect of low acute morphine doses (0.1–1.0 mg/kg i.v.). The development of analgesic tolerance to morphine was abolished by L-365,260, a selective cholecystokinin-B (CCKB) receptor antagonist [111].

He and coworkers [40] investigated the effect of systemic and local (i.pl.) loperamide on spinal nerve injury-induced allodynia in mice. They found that local and systemic loperamide inhibited allodynia assessed in the von Frey test. In this study, repeated treatments with different i.pl. doses of loperamide developed tolerance only to the local drug effect. On the other hand, systemic pretreatment caused local and systemic tolerance to loperamide; however, systemic morphine remained effective. Furthermore, they also measured the effect of local administration of DAMGO, which also induced analgesic tolerance. The development of tolerance to systemic loperamide was attenuated by naltrexone pretreatment but not by co-treatment with naloxone or naloxone-methiodide. The NMDA receptor antagonist MK-801 attenuated tolerance development to systemic loperamide, but it was not influenced by the glycine β-site antagonist MDL 105,519 [75]. Analgesic tolerance to local loperamide was not influenced by naloxone methiodide or MK-801. Additionally, they found a decrease in total MOR protein in L5 segment of the spinal cord after Seltzer nerve ligation (SNL), which was more pronounced in saline-treated animals than in morphine- or loperamide-treated groups. The Ser375 phosphorylation, which is important in desensitization and tolerance development, was also increased in the morphine-treated group but not in loperamide-treated group. In an in vitro model of loperamide tolerance—KCl-induced Ca^2+^ current in DRG neurons—they found that tolerance was developed after exposure to loperamide, which was attenuated by the DOR antagonist, naltrindole.

An interesting and promising approach to overcome the reduced levels of peripheral MOR expression during neuropathic pain is the viral vector delivery of the MOR via herpes simplex virus type 1 (HSV-1) [109]. Upon infection of mice with nerve injury, the immune reactivity of MORs significantly increased in epidermal nerve fibers in the plantar hind paw skin, in large and medium-diameter DRG cells and in lamina I–III of the dorsal lumbar spinal cord. Additionally, the properties of cutaneous afferents also changed upon infection. HSV-1 MOR inoculation in Aδ-fibers hindered the SNL-induced enhancement to suprathreshold stimulation, while the occurrence of C-fibers with spontaneous activity was reduced. Most importantly, HSV-1 MOR inoculation reversed mechanical allodynia and thermal hyperalgesia, and it also showed a leftward shift in loperamide- and morphine-induced analgesia in nerve-injured animals.

These findings raise important questions, such as: How does viral vector delivery of MOR in the periphery affect opioid-induced tolerance in the periphery or even in the CNS? Additionally, if the low MORs reserve in the periphery can be restored or further improved following nerve injury, the previously described peripherally acting opioid compounds with limited CNS penetration [34,47,60,62,65] might be able to achieve significant antinociception in neuropathic pain. Exploring these questions would be of interest to effectively treat neuropathic pain with opioids and to overcome opioid-induced analgesic tolerance (Table 2).

## 3. Drawbacks of Peripheral MORs Activation Related to Opioid Analgesic Tolerance

### 3.1. The Consequence of MORs Activation on Primary Sensory Neurons

All desirable and unwanted opioid side effects largely stem from the activation of MORs that are distributed either in the CNS or in the periphery [97]. A general assumption was that central analgesic tolerance is the consequence of a decrease in the central MORs response to opioid analgesics; however, the involvement of peripheral MORs in the development of central analgesic tolerance has also gained attention recently [112,113].

Recent data from Corder and co-workers showed that the activation of peripheral MORs (expressed by primary afferent nociceptors) is involved in the development of central analgesic tolerance to MOR agonists [114]. In addition, these receptors also have a crucial role in OIH. Namely, the deletion of these peripheral receptors eliminated both morphine tolerance and OIH. This finding was further supported by the pharmacological inhibition of MORs at peripheral sites. Methylnaltrexone, a peripherally acting opioid antagonist, abrogated the analgesic tolerance when co-administered with morphine, without diminishing the analgesic effect of morphine, in different pain models (perioperative and chronic pain models). These results indicate that the systemic co-administration of peripherally acting opioid antagonists with opioid analgesics that readily penetrate into the CNS might be a new clinical approach for the prevention of central analgesic tolerance development [114]. Earlier studies by Danysz and co-workers found that the co-administration of naloxone methiodide inhibited the development of morphine tolerance [77]. They investigated the effects of morphine in morphine-tolerant mice in the tail-flick test in different subspecies of mice [77]. Another strategy for central analgesic tolerance prevention might be the activation of other peripheral ORs (DORs or KORs). In the late 1990s, Walker and co-workers found that peripherally acting KOR agonists successfully alleviated pain symptoms in morphine-tolerant rats in a sciatic nerve injury model [115]. Although this way seems promising, only few peripherally acting compounds reached clinical studies up to this date [116].

Upon the chronic use of centrally acting opioids, anti-opioid systems also actively contribute to the development of opioid analgesia. On the other hand, drugs that inhibit anti-opioid systems (NMDA, CCK) have been long reported to abrogate analgesic tolerance [117,118,119,120]. These systems appear to have a crucial role in the development of peripheral opioid analgesic tolerance, although few data are available. In 2005, Danysz and co-workers proved the role of peripheral NMDA receptors in analgesic tolerance development in the tail-flick test in different subspecies of mice [77]. They used peripherally acting NMDA receptor/glycine B site antagonists (MRZ 2/596 and MDL), after proving their lack of CNS effects. Their results indicated that the peripheral blockade of NMDA receptor/glycine B sites can attenuate morphine analgesic tolerance.

In this regard, under neuropathic pain conditions, the peripheral MORs have been reported to be also decreased [121,122,123], but it seems that this change does not alter the development of central opioid analgesic tolerance, although it has not been thoroughly investigated so far. According to the above studies, the correlation between the change in the MORs reserve in primary sensory neurons and the development of central and peripheral opioid analgesia may be worth investigating. In contrast, a recent study by Klein and co-workers [109] demonstrated that the herpes simplex virus (HSV) MOR inoculation increased the analgesic activity of loperamide or morphine following systemic administration.

The development of tolerance is a highly complex mechanism that is still not fully understood. This complicated mechanism involves several different pathways, not just centrally, but also peripherally. Among the strategies that might hinder the development of central opioid analgesic tolerance are those affecting the downstream targets of opioid receptors, such as ATP-sensitive potassium (K_ATP_) channels. The activity of these channels can influence the efficacy of opioids and represent an important factor in tolerance development. Cole Fisher and co-workers demonstrated that the downregulation of SUR1 subtype K_ATP_ channels in the spinal cord and DRG potentiated the development of morphine tolerance and withdrawal syndrome in mice [124]. SUR1 agonists (diazoxide and NN-414) attenuated tolerance development. These results suggested that increasing neuronal K_ATP_ channel activity in the peripheral nervous system might be a viable option to alleviate central opioid analgesic tolerance and withdrawal syndrome. Several recent studies have outlined how peripheral MORs affect the central analgesic tolerance. Of importance is the consequence of the activation of peripheral MORs on the gut.

The above-mentioned strategies might potentially solve the development of central analgesic tolerance, but the addiction liability of centrally acting opioid analgesics remains unsolved.

### 3.2. The Role of MORs in the Gut Microbiota: Dysbiosis, Opioid Tolerance

In the past decades, a huge amount of data has been accumulated on the role of gut microbiota in the pathogenesis of various GI (irritable bowel syndrome, inflammatory bowel diseases, colorectal cancer), endocrine (obesity, diabetes), cardiovascular, and even neuropsychiatric diseases [125,126,127]. Based on recent findings [41,128,129,130,131], the development of opioid analgesic tolerance can also be added to the continuously increasing list of adverse events related to microbial alterations (dysbiosis) in the GI tract. This section provides a brief overview of opioid-induced dysbiosis and the data supporting the concept that microbial alterations contribute to analgesic tolerance. Notably, a detailed review by Mischel and co-workers [132] has been recently published, to which the reader is referred for further information and more extensive bibliography.

Regarding opioid-induced dysbiosis, to date, most of the data originate from preclinical studies, in which mice were exposed to morphine for different time periods [128,129,131,133,134,135]. However, in some experiments, other opioid agents were used, such as loperamide [136,137] or hydromorphone [138], and there are some common patterns in the microbial composition of animals irrespective of the type of opioid used, allowing to draw some general conclusions on the effect of opioids on microbiota. Moreover, some of these changes have also been observed in non-human primates [139], as well as in opioid user cirrhotic patients compared to those not on opioids [140], or in heroin addicts [141], further supporting the complexity and translational relevance of the results.

Although there are some variabilities between the findings of the different studies, which may also result from the various treatment protocols [135], the administration of opioids is in general accompanied by the expansion of *Staphylococcus* and *Enterococcus* genera within the *Firmicutes phylum* and by reduced abundance of the *Lactobacillus* genus. The decreased representation of the *Clostridia* class and in particular the contraction of the *Lachnospiraceae* and *Ruminococcaceae* families are also relatively consistent results. Another common finding in opioid-treated rodents is the expansion of the *Proteobacteria phylum* with increased abundances of *Enterobacteriales* and the genus *Sutterella*. In addition, various (and sometimes contradictory) changes within the *Bacteroidetes phylum* have been reported, from which the expansion of the *Porphyromonadaceae* and *Prevotellaceae* families appears in several reports. These effects depend on the activation of MORs, as morphine failed to induce dysbiosis in naltrexone-treated and MOR-KO animals [129,133,134].

It is noteworthy that several bacteria with increased abundance are considered to be potentially pathogenic, including *Enterococcus*, *Sutteralla,* and *Enterobacteriaceae* [142,143,144], whereas bacteria with decreased amount (*Lactobacillus*, *Lachnospiraceae*, *Ruminococcaceae*) have anti-inflammatory properties, and some *Lactobacillus* species are widely used probiotics [145,146]. Hence, opioid-induced microbial alterations can generate a pro-inflammatory milieu, which can compromise the gut epithelial barrier and allow luminal aggressive factors (bacteria, bile acids) to penetrate into the gut wall and trigger an immune response, further amplifying the initial inflammation. Gut inflammation in response to opioid administration is typically characterized by enhanced intestinal permeability, the activation of Toll-like receptor-2 (TLR-2), TLR-4, and elevated levels of various pro-inflammatory cytokines, including tumor necrosis factor-α (TNF-α), interleukin-1β (IL-1β), IL-6, IL-17) in the intestine, mesenteric lymph nodes, and remote organs [128,133,134,138].

Several lines of evidence suggest that opioid-induced dysbiosis contributes to the development of analgesic tolerance. Elimination of the gut microbiota, either by using germ-free mice or by treating conventionally raised animals with a broad-spectrum antibiotic cocktail, significantly attenuated tolerance development to chronic morphine in different in vivo assays [128,131]. By contrast, prolonged exposure to morphine induced tolerance in germ-free mice that had undergone fecal microbiota transplantation with samples obtained from conventionally raised mice [128]. Among tested antibiotics, vancomycin, a non-absorbable glycopeptide antibiotic that selectively eliminates Gram-positive bacteria, appeared to have the most prominent effect, as it was able to reduce morphine tolerance even alone, although not as effectively as in combination with other antibiotics [130,131]. Therefore, the expansion of distinct Gram-positive strains in response to opioid treatment may have a pivotal role in the development of analgesic tolerance. This concurs with the findings that the exposure of morphine promoted Gram-positive sepsis and the production of IL-17 in a TLR-2-dependent manner (which receptor recognizes components of the Gram-positive cell wall) [133,134]. Moreover, the genetic deletion of TLR-2 had a more pronounced inhibitory effect on the development of opioid tolerance than deletion of TLR-4 (which is primarily activated by the Gram-negative cell wall component lipopolysaccharide) [128]. Among the Gram-positive bacteria *Enterococcus* may be of particular relevance, as it blooms in the gut of opioid-treated animals [129,133,134,138] and can also be detected in the peritoneal organs of these animals due to impaired epithelial barrier and bacterial translocation [133]. Further evidence for the importance of this pathogen is that infection with *Enterococcus faecalis* augmented the development of morphine tolerance in mice [129].

The above-mentioned data provide clear evidence for the contribution of opioid-induced dysbiosis to the development of analgesic tolerance; however, research is still going on to identify the underlying mechanisms. In addition, dysbiosis following opioid treatment is typically characterized by the expansion of potentially pathogenic bacteria, which may trigger epithelial damage and intestinal inflammation. This inflammatory reaction is likely to be a key factor in tolerance development, and based on some evidence, it is effectively reduced by antibiotic treatment. For example, whereas the colonic mucosa of morphine-treated animals was characterized by histological damage and elevated levels of the pro-inflammatory cytokine IL-1β, these changes were largely prevented by an antibiotic cocktail [128]. Similar results were found by Zhang et al. [125,128], who reported reduced damage and lower tissue levels of TNF-α, IL-1β, and IL-6 in the ileum of mice treated with both morphine and antibiotics, compared to only morphine-treated animals.

However, it is still unclear exactly which sites of the pain pathway are affected primarily by the intestinal inflammation. Some studies point to the importance of inflammation-induced alteration in the excitability of DRG neurons. As mentioned before, the activation of MORs expressed by these neurons have been reported to contribute significantly to opioid tolerance via initiating multiple downstream events in pain conducting pathways [114]. In addition, it is well-established that inflammation of the GI tract induces hyperexcitability in nociceptive DRG neurons [147], and it was recently demonstrated that experimental colitis induced by 2,4,6-trinitro-benzene sulfonic acid (TNBS) enhanced the development of morphine tolerance in mice [148]. Therefore, it is plausible that opioid-induced dysbiosis, accompanied by epithelial injury and tissue inflammation, results in analgesic tolerance at least due in part to the altered activity of primary afferents. This assumption is supported by the findings that the inhibitory effect of antibiotic treatment on the development of opioid analgesic tolerance can also be demonstrated in DRG neurons [130,131]. Namely, in animals treated with both morphine and an antibiotic cocktail (or vancomycin), the inhibitory effect of an acute morphine challenge on the excitability of DRG neurons remained unaltered, in contrast to animals treated chronically only with morphine, where due to cellular tolerance development, acute morphine administration failed to affect the neuronal activity. Besides altering the functions of primary afferents, opioid-induced dysbiosis and systemic inflammation may induce tolerance in nociceptive circuits of the central nervous system as well. A growing body of evidence links enhanced central immune signaling and increased neuronal excitability to the development of opioid tolerance [149], and bacterial components and systemic inflammatory mediators originating in the gut may evoke central neuronal responses as well, especially if the blood–brain barrier is compromised due to chronic opioid exposure [150].

The question arises: Which MOR-mediated opioid effects are mainly responsible for the observed microbial alterations? Different opioids, including morphine, can suppress the activity of basically all cell types involved in innate and adaptive immunity [151]. Since the immune system is one of the major determinants of the microbiota composition [152], opioid-induced alterations in the intestinal immune functions may have a significant role in the pathogenesis of dysbiosis. Indeed, in the study of Banerjee and co-workers [134], morphine failed to induce dysbiosis in severely immunocompromised mice, indicating that the effects of morphine on the microbiome depend on immune modulation. The opioid-induced inhibition of GI motility is also likely to be involved in dysbiosis [41]. The bidirectional interaction between motility and microbiota is well-known [136,153], and as mentioned above, loperamide-induced dysbiosis resembles in some aspects that caused by morphine [136,137]. In addition, changes in bile acid metabolism due to opioids may also contribute to the consequence of dysbiosis [134,139].

The fact that opioid-induced dysbiosis has a significant role in the tolerance development to the analgesic effect of opioids has several important clinical implications. First of all, the modulation of the microbiota composition either directly (with probiotics or GI-restricted antibiotics) or indirectly (with dietary manipulation or promoting the GI peristalsis) may provide a novel approach to prevent or reduce opioid tolerance. Some of these manipulations have already been proven to be effective in animal experiments—for example, treatment with vancomycin (as mentioned before), administration of the probiotic VSL#3 [128], or oral treatment with butyrate (which has both anti-inflammatory and motility promoting effects) [41]. Certainly, clinical studies are warranted to confirm the tolerance-reducing property of these treatments in humans and to find the best strategies with the lowest risk–benefit ratio. On the other hand, the phenomenon of dysbiosis-related analgesic tolerance suggests that even peripherally restricted opioids may lose their analgesic properties in the case of chronic administration due to their GI effects. Whether and to which extent the peripherally restricted opioids that have been recently proved to produce peripheral analgesia can alter the gut microbiota, and whether this dysbiosis correlates with peripheral analgesic tolerance, are issues that warrant further investigation.

## 4. Summary and Conclusions

Many attempts have been made to minimize the development of opioid analgesic tolerance in order to avoid the consequence of opioid dose escalation. These studies focused largely on the central mechanisms underlying opioid analgesic tolerance. It is worth mentioning that the worldwide opioid crisis is the result of the use of centrally acting opioid analgesics for controlling pain. Targeting the functional peripheral ORs is an alternative strategy to provide adequate pain relief with less health risks that are related to the current opioid epidemic due to centrally acting opioids. A huge body of evidence indicates the development of central analgesic tolerance to all opioid analgesics. On the other hand, few studies have been published in relation to peripheral analgesic tolerance of opioids. Herein, the reviewed data suggest that the development of peripheral analgesic tolerance is largely dependent on the pain entity, animal pain models and the route of administration, locally versus systemically. Apparently, there is no consensus on the occurrence, magnitude, and the time course regarding the development of analgesic tolerance at peripheral sites in animal models of acute thermal, inflammatory, and neuropathic pains. There are promising approaches to avoid analgesic tolerance, such as designing opioid agonists with limited CNS penetration. However, evaluations of these compounds in relation to peripheral analgesic tolerance have not been fully carried out yet. In addition, the question can be raised as to whether these compounds produce significant peripheral analgesic effects upon long-term treatment. Opioid analgesic tolerance is developed as a consequence of the reduction in the number of available ORs for agonists on the cell surface in key points of pain pathways including the periphery. Consequently, novel opioids of high efficacy and limited CNS penetration might be of clinical value, because the risk of CNS-mediated side effects such as respiratory depression, addictive liability, and overdose incidence can be decreased. Despite the published data on the drawbacks of targeting peripheral MORs in the development of central analgesic tolerance, convincing pharmacological and genetic (the inclusion of peripheral MORs) approaches related to peripheral analgesia and tolerance support such claims. Additionally, restoring the opioid-induced dysbiosis might have an important potential in clinical practice. Finally, the success in developing opioid agonists devoid of central opioid side effects while inducing effective, peripherally mediated analgesia would be a great advance in clinical pain management, together with decreasing addictive liability and overdose deaths.

## Figures and Tables

**Table 1:** Examples of MOR agonists from the class of morphinans with limited CNS penetration and peripheral analgesic effect.

Structure	R_1_	R_2_	R_3_	Compound	Ref.
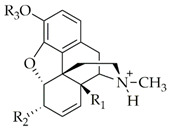	H	OSO_3_^−^	H	Morphine-6-*O*-sulfate	[73]
	OCH_3_	OSO_3_^−^	H	14-*O*-methylmorphine-6-*O*-sulfate	[62]
	H	OSO_3_^−^	CH_3_	Codeine-6-*O*-sulfate	[60]
	OCH_3_	OSO_3_^−^	CH_3_	14-*O*-methylcodeine-6-*O*-sulfate	[65]
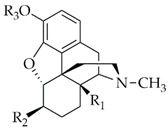					
	OCH_3_	HNCH_2_COOH	H	14-*O*-methyloxymorphone-6β-glycine (HS-731)	[34,35]


**Table 2:** Summary of evaluated compounds for peripheral opioid analgesic tolerance in different pain models.

Pain Model	Assay and Species	Test Compound	Route of Administration	Tolerance Induction	Main Findings	Ref.
Acute thermal	MTF	Morphine M6G	Tail injection	s.c. morphine	High grade tolerance	[74]
Acute thermal	MTF	Morphine M6G DAMGO	DMSO solution immersion	topical morphine	High grade tolerance	[75]
PGE2-induced pain	RS in rat	DAMGO	i.pl. injection	i.pl. injection DAMGO	Tolerance, withdrawal hyperalgesia	[76,86]
Bradykinin-induced flexion reflex	mouse	DAMGO U69,593 DESLET morphine	i.pl. infusion	s.c. morphine	No tolerance to local effect	[89,90]
Bradykinin-induced flexion reflex	mouse	Morphine	i.pl. infusion	i.pl. morphine	Tolerance to local effect	[91]
Carrageenan-induced inflammation	RS in rat, spinal c-Fos	Morphine	i.pl. (and i.v.) injection	s.c. morphine 3 days 2x	Tolerance to i.pl. or i.v.	[87]
CFA-induced inflammation	Plantar, RS, von Frey in mouse	Morphine	s.c.	morphine pellet 3 days	Higher grade tolerance on CFA treated paw than noninflamed	[99]
CFA-induced inflammation	RS in mouse	Morphine Fentanyl Bup. DPDPE U50488H CRF	i.pl.	morphine pellet 3 days	E_max_↓ in all case (exc. buprenorphine) and rightward shift (exc. buprenorphine and fentanyl)	[100]
CFA-induced inflammation	RS in rat	Fentanyl	i.pl.	s.c. morphine (4 days 2x)	Tolerance developed only in absence of inflammation	[68]
SNL	von Frey in mouse	Loperamide	i.pl./s.c.	i.pl./s.c. loperamide	i.pl. loperamide caused tolerance only on i.pl., caused both for i.pl. and s.c.; s.c. morphine remained effective	[40]
CFA-induced inflammation	Hargreaves assay in rat	Loperamide–oxymorphindole	topical (left hind paw)	twice a daily for 3 days	No tolerance to local effect	[102]

MTF: mouse tail-flick; Bup: buprenorphine; RS: Randal–Selitto; SNL: spinal nerve ligation; i.pl.: intraplantar; s.c.: subcutaneous; i.v.: intravenous; CFA: complete Freund’s adjuvant; CRF: corticotrophin releasing factor; DMSO: dimethyl sulfoxide.
